# Comparison of polybrominated diphenyl ethers (PBDEs) and polychlorinated biphenyls (PCBs) in the serum of hypothyroxinemic and euthyroid dogs

**DOI:** 10.7717/peerj.3780

**Published:** 2017-09-12

**Authors:** Grace Lau, Kyla Walter, Philip Kass, Birgit Puschner

**Affiliations:** 1 Department of Molecular Biosciences, University of California, Davis, CA, United States of America; 2 Department of Population Health and Reproduction, University of California, Davis, CA, United States of America

**Keywords:** PBDEs, PCBs, Thyroid hormones, Canine, Hypothyroidism, Hypothyroxinemia

## Abstract

**Objective:**

To determine the profile of 14 polybrominated diphenyl ethers (PBDEs) and 23 polychlorinated biphenyls (PCBs) in serum of domestic canines and whether this was predictive of thyroid hormone status.

**Samples:**

Serum samples were collected from 51 client-owned dogs visiting the University of California Davis William R. Pritchard Veterinary Medical Teaching Hospital during 2012 to 2016 for routine appointments. Fifteen dogs were diagnosed with hypothyroxinemia while 36 were euthyroid.

**Procedures:**

Concentrations of PBDEs and PCBs in canine serum samples were measured by gas chromatography mass spectrometry. Logistic regression analysis was used to determine the association between the presence/absence of canine hypothyroxinemia and the serum concentration of individual PBDE or PCB congeners.

**Results:**

The median concentrations of total PBDE and PCB congeners in the hypothyroxinemic group were 660 and 1,371 ng/g lipid, respectively, which were higher than concentrations detected in the control group. However, logistic regression analysis determined that current concentrations of PBDEs and PCBs in canines were not significantly associated with hypothyroxinemia. BDE 183 was the only congener showing near significance (*p* = 0.068).

**Conclusions:**

PBDE and PCB congeners were detected in all canine samples confirming ongoing exposure to these pollutants. Because household dogs share the human environment, they may serve as biosentinels of human exposure to these contaminants.

## Introduction

The World Health Organization has reported that thyroid disorders are amongst the most prevalent of medical conditions (WHO/UNEP, 2013), with primary hypothyroidism being the most common endocrinopathy in humans and dogs (Milne & Hayes, 1981; Scott-Moncrieff, 2015). There is an increasing concern that environmental chemicals contribute to the prevalence of thyroid hormone disorders (Boas, Feldt-Rasmussen & Main, 2012). This is driven largely by epidemiological and experimental evidence demonstrating that environmental chemicals may influence thyroid homeostasis by interfering with thyroid hormone signaling and regulation (Boas, Feldt-Rasmussen & Main, 2012; Carpenter, 2006; Kim et al., 2014; WHO/UNEP, 2013). Polybrominated diphenyl ethers (PBDEs) and polychlorinated biphenyls (PCBs) are two structural classes of persistent organic pollutants for which there is strong evidence of their potential to disrupt thyroid hormone function (Boas, Feldt-Rasmussen & Main, 2012; Pearce & Braverman, 2009; Zoeller, 2005). The compounds’ potential toxicity is concerning because their chemical stability and lipophilic nature allows them to persist in the environment and bioaccumulate. Given the importance of thyroid hormones in animal and human physiology (Scott-Moncrieff, 2015), evaluating the relationship between the body burden of PBDEs and PCBs to thyroid hormone status is reasonable. Furthermore, several endocrine diseases of humans, including diabetes mellitus and hypofunction syndromes of the thyroid, occur similarly in dogs and cats (Rijnberk, Kooistra & Mol, 2003). Studies have also shown histologically that thyroiditis in beagles resembled Hashimoto’s disease in humans (Beierwaltes & Nishiyama, 1968; Gosselin, Capen & Martin, 1978). Therefore, a canine animal model may prove helpful for evaluating the role of environmental determinants of thyroid disease.

Human exposure to thyroid hormone disrupting chemicals is of particular concern; however, biomonitoring studies are difficult. Human studies that involve biological sample collection require extensive procedures to obtain necessary informed consent, and face challenges with enrollment of volunteers and subject privacy. Because of factors like chronic low-dose exposure, multiple exposure routes, long latency periods, and non-specific health outcomes, conducting the appropriate human assessment to demonstrate causality is challenging (Backer et al., 2001). A number of studies have used pet dogs as sentinels for environmental health to associate contaminants with certain disease states such as household asbestos exposure with mesothelioma, household application of herbicides 2,4-D with malignant lymphoma, and environmental tobacco smoke with lung cancer (Backer et al., 2001), with only a few publications characterizing PBDE and PCB exposure assessments of canines (Ali et al., 2013; EWG, 2008; Mizukawa et al., 2016; Ruiz-Suarez et al., 2015; Storelli et al., 2009; Venier & Hites, 2011).

Domestic pets, such as cats and dogs, share much of their environment with humans, thus they may serve as biosentinels of potential human health hazards by providing data on the contaminants within a defined area and serving as a proxy of human exposure to these environmental contaminants (Venier & Hites, 2011; Walter et al., 2017). Even though pets and their owners share the same air, water, and housing, pets are usually free of lifestyle factors that may confound associations with true risk factors in humans, such as tobacco use, alcohol and caffeine consumption, poor diet, insufficient physical activity, and low social class (Schmidt, 2009). Studies have reported over 450 diseases in the domestic dog, with approximately 360 diseases being analogous to human diseases (Shearin & Ostrander, 2010). Further analyses found that the genome sequence in dogs were more homologous in sequence to humans than mice. Dogs respond to many toxic chemicals similarly to humans, developing diseases by comparable pathogenic mechanisms with shorter latency periods. One example is the use of dogs as an animal model for toxicological studies of lead exposure (Schmidt, 2009). Canine cancers have also been recognized as appropriate models for human cancer studies, particularly non-Hodgkin’s lymphoma (Takashima-Uebelhoer et al., 2012). The cytochrome P450 enzymes are important catalysts in metabolism of xenobiotics and chemical toxicology. When comparing different species, including dogs, rabbits, and monkeys, to man as potential animal models, no species was similar for all P450 enzyme activities (Bogaards et al., 2000). Between each species, only some enzymes could be considered similar to man. For instance, CYP2D15 in dogs appears to have enzymatic activity similar to human CYP2D6 and may be useful in studies based on metabolism mediated by this enzyme (Zuber, Anzenbacherova & Anzenbacher, 2002). Therefore, knowledge of body burdens of chemicals in domestic canine populations may be useful for predicting exposures that pose potential risks to humans (Schilling et al., 1988; Storelli et al., 2009).

The work described in this study tests the hypothesis that serum collected from pet dogs will have detectable concentrations of PBDE and PCB congeners that are associated with thyroid dysfunction. Additionally, the aim of this study was to determine the profile of PBDEs and PCBs in dogs and to evaluate whether this was predictive of hypothyroxinemia.

## Materials and Methods

### Study population

Serum samples were obtained from client-owned dogs presented to the William R. Pritchard Veterinary Medical Teaching Hospital (VMTH), School of Veterinary Medicine, University of California Davis (Davis, CA, USA) during 2012 to 2016 for appointments and stored at −20 °C prior to analysis. Various breeds, such as Labradors, Border Collies, and Terriers, were included in this study. Sex, age, weight, and breed of the dogs are summarized in Table S1. A diagnosis of hypothyroidism was based on consistent clinical signs observed by a VMTH clinician and total serum T4 concentrations measured below the reference range of 1.0–3.2  µg/dl. Additional thyroid diagnostics, including serum TSH, TSH stimulation tests, thyroid biopsies or radioactive pertechnetate uptake studies were not performed. Therefore, we referred to the dogs with low serum total T4 levels as hypothyroxinemic. Both newly diagnosed cases of canine hypothyroxinemia and dogs receiving treatment for previously diagnosed hypothyroidism were included in the study. Control samples were collected randomly from dogs determined to be free of canine hypothyroidism based on the absence of clinical signs of hypothyroidism or any other endocrine disease. In total, 15 hypothyroxinemic dogs and 38 euthyroid control dogs were enrolled in this study. Two dogs from the control group were excluded from the analysis; one dog was diagnosed with hyperthyroidism and the age was unknown in the other. VMTH protocols were followed to obtain written consent from all owners to permit the use of their dogs’ samples in this study.

### Extraction of analytes

Samples were thawed on ice prior to extraction. Details of the extraction of samples for PBDE and PCB analytes have been described previously (Lin, Pessah & Puschner, 2013). In brief, 0.25 ml aliquots of serum were transferred into Eppendorf tubes for total lipids analysis. To the remaining serum sample of 0.25 –0.5 ml aliquots, internal standards (Cambridge Isotope Laboratories, Inc., Tewksbury, MA, USA) 1ng ^13^C_12_ labeled 2,3′,4,4′,5-penta BDE (^13^C_12−_ BDE-118) and 1 ng ^13^C_12_ labeled 2,2′,3′,4,5-pentachlorobiphenyl (^13^C_12_-PCB-97) were added before mixing with 0.5 ml of pure formic acid (98+%, ACROS Organics™, Fisher Scientific, Hampton, NH, USA) vortexing for 1 min, and gravimetric filtration through Solid Phase Extraction columns (Waters Oasis HLB SPE cartridges; Milford, MA, USA). Columns were previously conditioned with analytical grade methanol and ultrapure water with 1% formic acid. For further clean-up, silica cartridges (Sep-pak^®^ Light Silica cartridges; Waters, Milford, MA, USA) were placed beneath SPE columns and analytes were eluted with three washes of 3 ml analytical grade dichloromethane under vacuum. Eluents were collected in disposable glass tubes containing 100  µL of 1 ng/ml Mirex (PESTANAL^®^, analytical standard; Sigma-Aldrich, St. Louis, MO, USA) as an internal standard to evaluate instrument performance. Samples were dried under a gentle stream of nitrogen (Organomation Associates, Inc., Berlin, MA, USA) in a water bath (40 °C), before being reconstituted in 100  µl of pure isooctane (99%, HPLC Grade, Fisher Chemical; Hampton, NH, USA). The sample was then transferred into an auto-sampler vial for analysis. Internal standards (Cambridge Isotope Laboratories, Inc., Tewksbury, MA, USA) ^13^C_12−_ BDE-118 and ^13^C_12_-PCB-97 were used throughout the extraction and analytical procedures. Six-point calibration curves consisting of PBDE and PCB concentrations of 0.1, 0.2, 0.8, 2, 4, and 10, ng/ml were prepared by adding PBDE and PCB analytical standards (Accustandard, Inc., New Haven, CT, USA) to 0.5 ml of control human serum (DDC Mass Spect Gold^^®^^, MSG 3000; Golden West Biologicals Temecula, CA, USA). Calibration samples were processed following the same extraction method as samples from enrolled dogs.

### Instrument analysis

Samples extracts were analyzed using gas chromatography coupled with triple quadruple mass spectrometry (GC/MS/MS, Scion TQ triple quadruple mass spectrometer; Bruker, Fremont, CA, USA) for BDE-17, -28, -47, -49, -52, -66, -85, -95, -99, -100, -136, -153, -154, and -183, and PCB-11, -28, -52, -66, -77, -84, -91, -95, -101, -118, -131, -132, -135, -136, -138, -149, -153, -174, -175, -176, -180, -196, and -202 following a previously published method (Lin, Pessah & Puschner, 2013). All analytes were quantified using the 6-point calibration curve with standard concentrations ranging from 0.1–10 ng/ml. Calibration curves were weighted 1/x. Following analysis, concentrations of analytes extracted from samples less than 0.5 ml of serum were corrected for volume. The lower limit of detection (LOD) and quantification (LOQ) were estimated based on a signal-to-noise ratio of 3:1 for the LOD and 10:1 for the LOQ. For statistical analysis, a non-detected congener was assigned a value of the corresponding LOD divided by $ \frac{1}{2} $.

### Quality control

The accuracy of the method was assessed using three quality control (QC) samples of human control serum (DDC Mass Spect Gold^^®^^, MSG 3000; Golden West Biologicals Temecula, CA, USA) fortified with all PBDEs and PCBs at concentrations of 0.1, 0.8 and 4 ng/ml (Table S2). QC samples were prepared following the same extraction method as described for the canine samples and analyzed in parallel with each group of canine samples. For each group of samples processed and analyzed, the determined concentration of each PBDE and PCB congener in the QC samples, as quantified by the standard curves, was required to fall within 70 to 120% of the known concentration of the individual congener for the data to be included in the final analysis (see Table S2). These parameters were borrowed from a study by Roszko et al. (2012) investigating PBDEs and PCBs in vegetable oil and established by the European Union for analytical quality control for pesticide residue analysis in food and feed (European Commission, 2015; Roszko et al., 2012). In addition, accuracy and precision were assessed using the certified SRM^^®^^1958 NIST reference standard (SRM^^®^^1958; National Institute of Standards and Technology reference standard (NIST), Gaithersburg, MD, USA) as previously described (Lin, Pessah & Puschner, 2013). Laboratory contamination and cross-contamination were checked by preparing and analyzing the ultra-pure water samples following the exact same procedures as used for samples from enrolled dogs. A procedural blank consisting of isooctane was run with every batch of samples. No contamination was detected when analyzing these samples by GC-MS/MS. The analytical laboratory participates in the Artic Monitoring and Assessment Program (also known as AMAP Ring Test of Persistent Organic Pollutants in Human Serum) (CTQ, 2014) for PBDE and PCB analyses and consistently showed excellent performance for PBDE congener analysis with a −2<Z’-score <2.

### Lipid content determination

Total lipid content was calculated after determination of total cholesterol (TC) and total triglycerides (TG) in each canine sample as quantified by the UC Davis Health System Department of Pathology and Laboratory Medicine using standard clinical chemistry enzymatic methods (Allain et al., 1974; Bucolo & David, 1973). The TC and TG concentrations were used to calculate the total lipids (TL) for each sample using the following equation by Phillips:

\begin{eqnarray*}TL=(2.27)\ast TC + TG + 62.3~(\mathrm{mg}/\mathrm{dl})~~\text{(Bernert et al. 2007)}. \end{eqnarray*}

### Statistical analysis

The median values and the 10th and 90th percentiles of 14 PBDE and 23 PCB congeners in canine serum samples were determined. Logistic regression was used to analyze the association between the presence/absence of canine hypothyroxinemia and the serum concentration of individual PBDE or PCB congeners. Based on an investigation by Milne & Hayes (1981), results indicated that potential risk factors for hypothyroidism in dogs include breed, sex, age, and gonadal status (Milne & Hayes, 1981). Thus, the association of age, sex, weight, and breed on hypothyroxinemia was investigated using exact Mann–Whitney and exact chi-square tests of homogeneity, respectively, to determine whether these variables should be controlled for in the logistic regression models (Table S3). To account for the various breeds, an online source, *The Kennel Club* (kennelclub.org.uk), was used to categorize the breeds into small, medium, and large sizes. For statistical purposes, small and medium sized breeds were later categorized together. Because the majority of the study population was already neutered or spayed, gonadal status was not included as a confounding variable. Lipid concentrations were compared between groups using exact Mann Whitney test (Table S3). Additionally, least squares linear regression model was used to evaluate the relationship between total lipids and each PBDE and PCB congener (Table S4).

A logistic regression model, including congener concentration and age as continuous (linear) variables, was used to analyze the association of a given congener concentration with the odds of hypothyroxinemia while controlling for the influence of age. Results are reported as odds ratios and 95% confidence intervals (CI) (Table S5). The initial statistical analysis was done using logistic regression in STATA data analysis and statistical software (Stata IC/13; StataCorp LP, College Station, TX, USA) which uses the standard maximum likelihood-based estimator. Due to the small sample size, any congeners which yielded *p*-values less than 0.15 were subsequently analyzed by exact logistic regression using LogXact statistical software (Cytel Software Corporation, Cambridge, MA, USA). *P*-values < 0.05 were considered statistically significant.

## Results

### Study population characteristics

A total of 15 hypothyroxinemic and 36 euthyroid control dogs were included in the study. The exact Mann–Whitney test, exact chi-square test, and logistic regression analysis indicated that age, sex, weight, and size were not significantly different between the control and hypothyroxinemic groups (*p* = 0.093; *p* = 0.203; *p* = 0.536; *p* = 0.757, respectively; Table S3); thus sex, weight, and size were not included in the logistic regression analyses. However, studies in Northern Europe, Japan, and USA have found the prevalence of hypothyroidism to be higher in elderly canines. In addition, PCB and PBDE concentrations increase with age (Glynn et al., 2003; Sjödin et al., 2008) Therefore, age was controlled for in logistic regression analyses to assess the influence of PBDE and PCB congener concentration on the risk of hypothyroxinemia (WHO/UNEP, 2013). The median age of hypothyroxinemic dogs in the study group was 10 years (5th–95th percentile, 6.4–12), compared to the median age of eight years (5th–95th percentile, 3.5–13.6) in the control group (Table S3).

### PBDE and PCB concentrations

**Figure 1:**
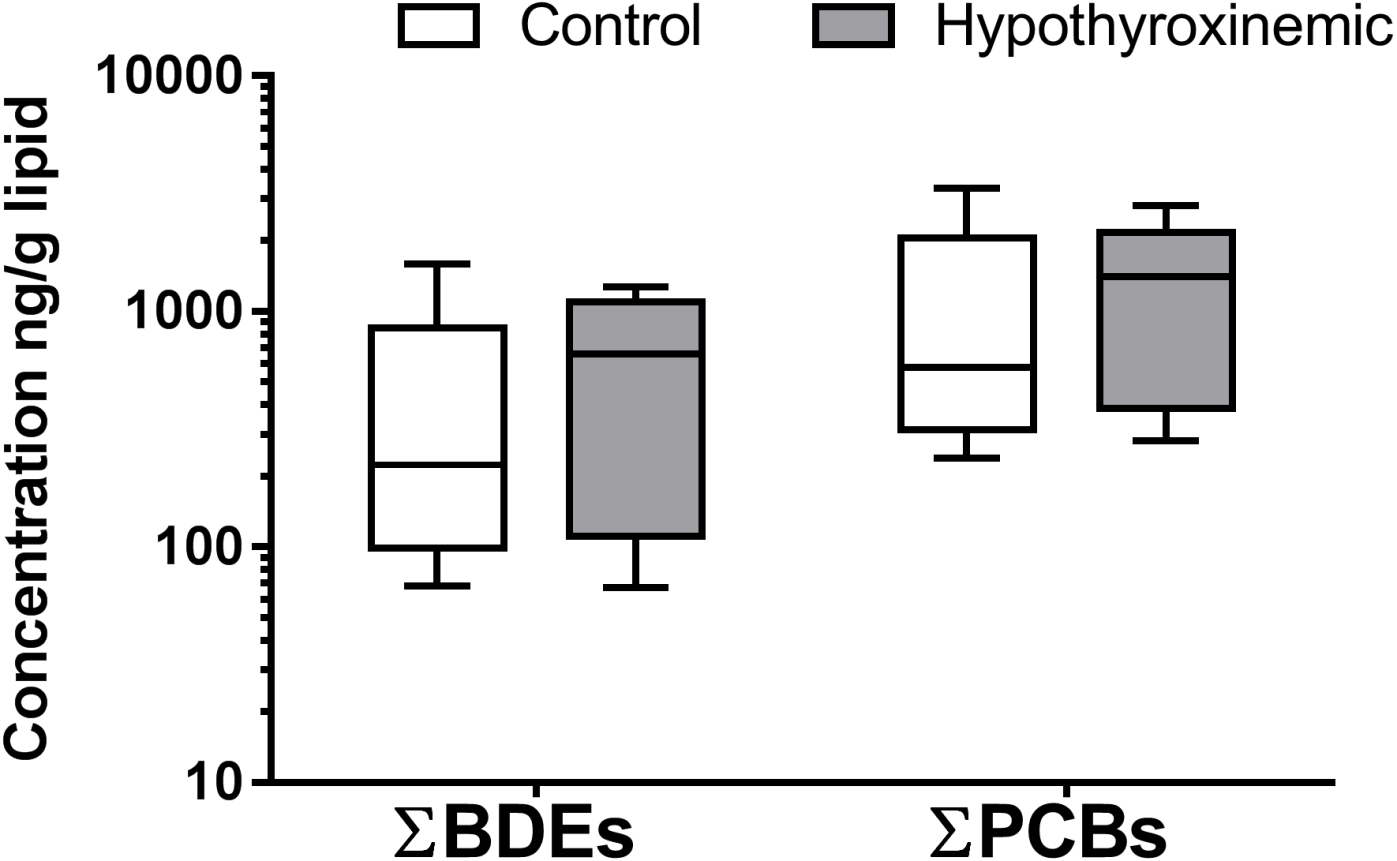
Box and whiskers diagram of ΣPBDEs and ΣPCBs concentrations in canine samples. The distribution of measured concentrations are presented as ng/g lipid for control, *N* = 36, (white boxes) and hypothyroxinemic, *N* = 15, (gray boxes) canine samples. The upper and lower boundaries of the boxes represent the 75th and 25th percentiles, and the line within the boxes indicates the median value. The upper whiskers show the 90th and the lower whiskers the 10th percentile. Not all PBDEs and PCBs were detected above LOQ in all animals. For statistical purposes, values found below the LOD were assigned a value of LOD/2.

The serum concentrations of 14 PBDE and 23 PCB congeners were determined and normalized to the total lipid concentration for each canine sample. The total lipids did not vary significantly between the hypothyroxinemic and control dog groups (*p* = 0.767; Table S3). Statistics also showed no significant correlation between total lipids and PBDE and PCB congener concentrations (Table S4). Based on previous data showing that lipid normalization can account for individual differences that occur with feeding schedules (Phillips et al., 1989) lipid corrected data was used to account for canine variability in the timing of blood collection and differences that might occur based on energy balance disruption in a hypothyroid canine. The distribution of ΣPBDEs and ΣPCBs for the hypothyroxinemic and control canine samples are shown in Fig. 1. The mean and median values of the ΣPBDEs were higher in the hypothyroxinemic group of canine samples compared to the control group. The mean (±SE) and median (10–90 percentiles) values of ΣPBDEs were 523.06 ng/g lipid (± 547.88 ng/g lipid) and 222.64 ng/g lipid (75.27–1555.16 ng/g lipid) for the control group compared to 660.16 ng/g lipid (± 457.33 ng/g lipid) and 660.02 ng/g lipid (70.70–1226.30 ng/g lipid) for the hypothyroxinemic group. Similarly, the mean and median values of the ΣPCBs were higher in the hypothyroxinemic group of canine samples compared to the control group. The mean (±SE) and median (10–90 percentiles) values of ΣPCBs were 1215.95 ng/g lipid (± 1238.28 ng/g lipid) and 576.36 ng/g lipid (238.09–3213.98 ng/g lipid) for the control group compared to 1371.23 ng/g lipid (± 968.73 ng/g lipid) and 1410.87 ng/g lipid (336.56–2550.27 ng/g lipid) for the hypothyroxinemic group. However, these observations were not statistically supported by logistic regression analysis (∑PBDEs *p* = 0.559, ∑PCBs *p* = 0.873).

**Figure 2:**
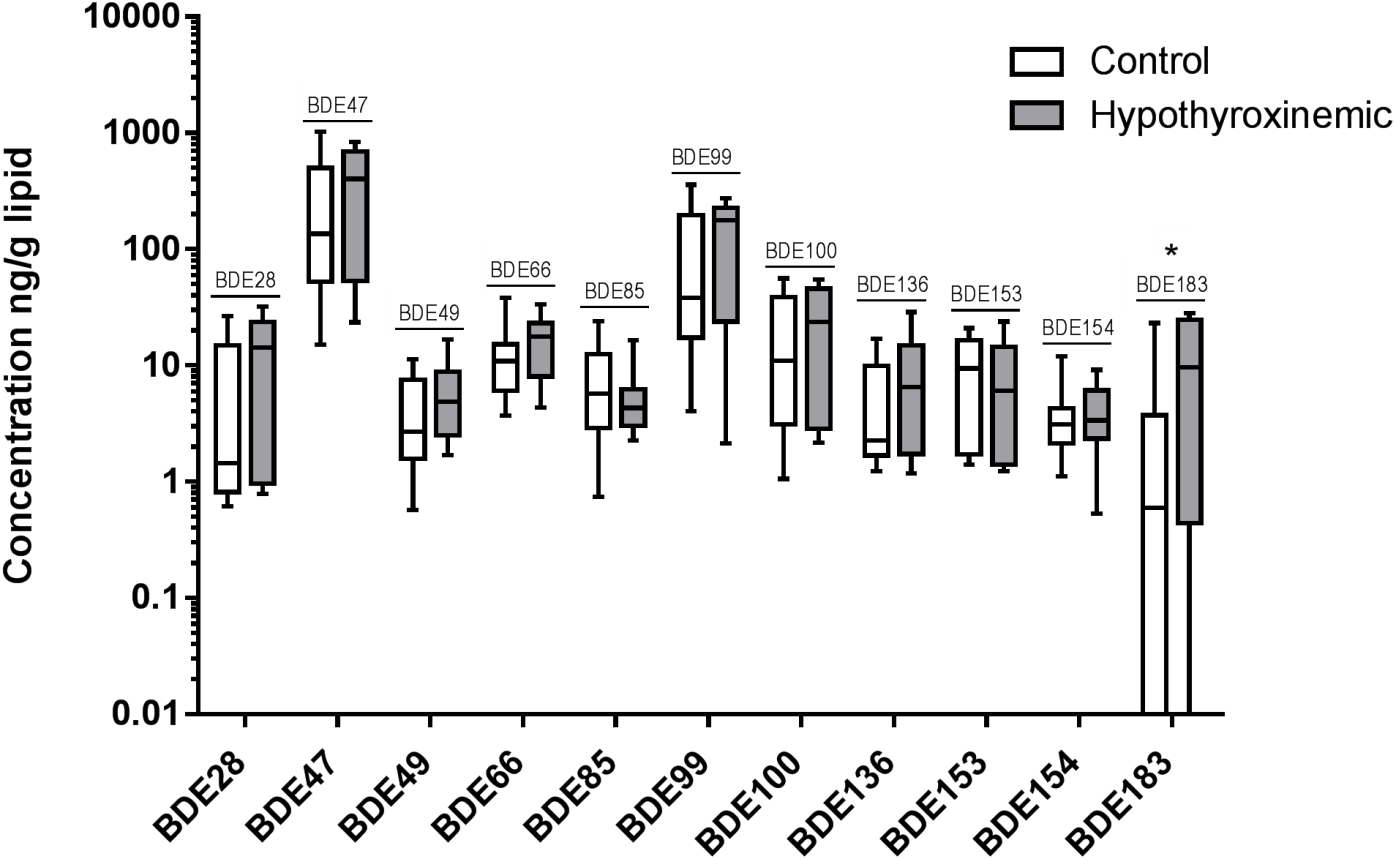
Box and whiskers diagram of individual PBDE congener concentrations in canine samples. The distribution of measured concentrations are presented as ng/g lipid for control, *N* = 36, (white boxes) and hypothyroxinemic, *N* = 15, (gray boxes) canine samples. The upper and lower boundaries of the boxes represent the 75th and 25th percentiles, and the line within the boxes indicates the median value. The upper whiskers show the 90th and the lower whiskers the 10th percentile. Not all PBDEs and PCBs were detected above LOQ in all animals. For statistical purposes, values found below the LOD were assigned a value of LOD/2. * *p*-value close to significance (≤ 0.05) in exact logistic regression controlling for age of canine study participants.

**Figure 3:**
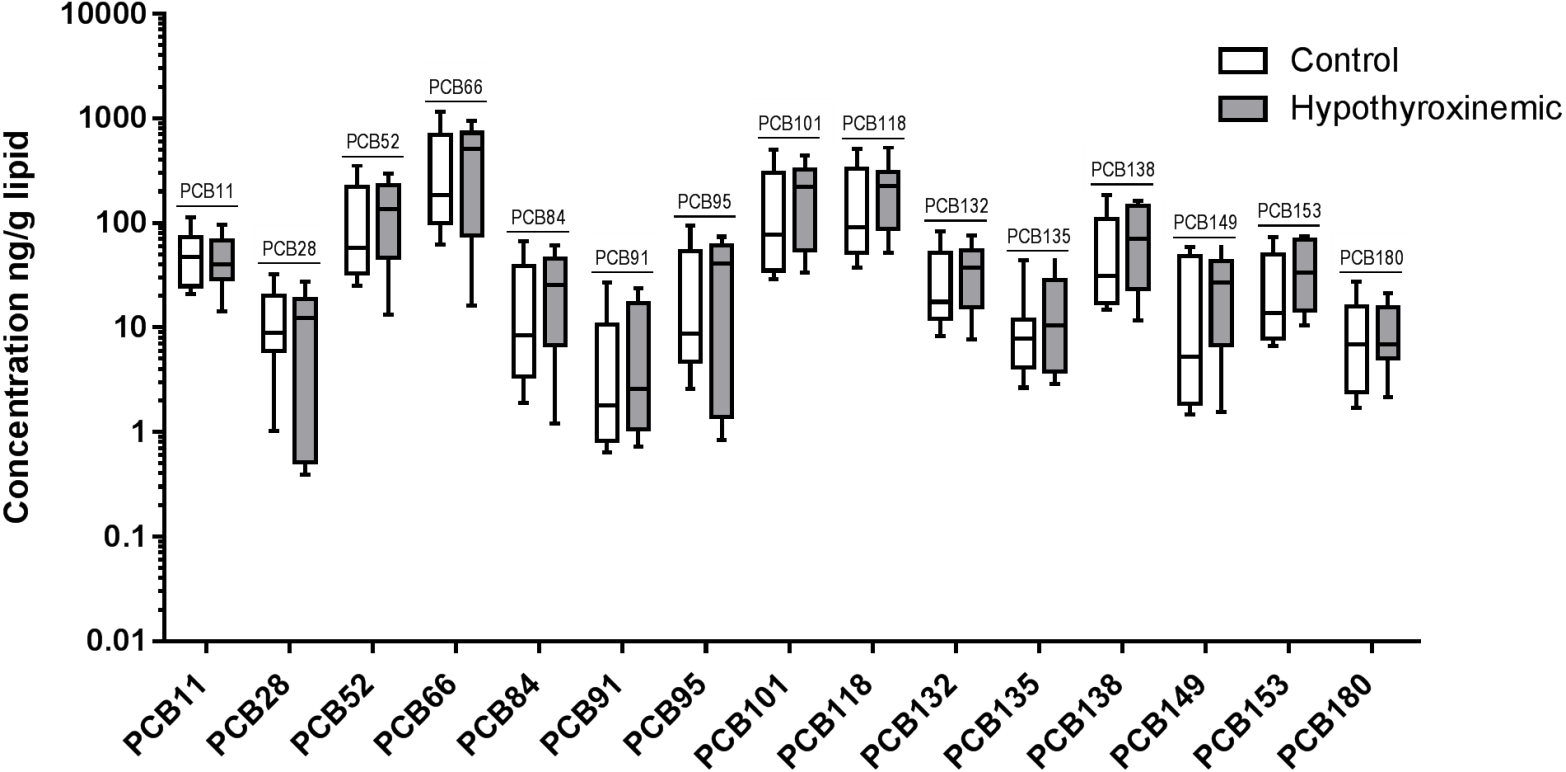
Box and whiskers diagram of individual PCB congener concentrations in canine samples. The distribution of measured concentrations are presented as ng/g lipid for control, *N* = 36, (white boxes) and hypothyroxinemic, *N* = 15, (gray boxes) canine samples. The upper and lower boundaries of the boxes represent the 75th and 25th percentiles, and the line within the boxes indicates the median value. The upper whiskers show the 90th and the lower whiskers the 10th percentile.

The distribution of individual PBDE and PCB congener concentrations measured (ng/g lipid) in the hypothyroxinemic and control groups are shown in Figs. 2 and 3. All congeners showed a large variation in serum concentrations, all of which were right skewed; whiskers on the box plot represent the 10th and 90th percentiles. Congeners were only included in the logistic regression analysis if the detection frequency was ≥ 20%; therefore, BDE 17, BDE 52, PCB 174, PCB 196, and PCB 77 were excluded. For BDE 17, a concentration of 0.54 ng/g lipid was detected in only one dog from the hypothyroxinemic group. For PCB 77, a concentration of 6.25 ng/g lipid was detected in only one dog from the control group. BDE 95, PCB 131, PCB 136, PCB 175, PCB 176, and PCB 202 were not detected in any canine sample.

**Table 1:** Median concentrations (range: 10^th^–90^th^ percentiles) of PBDEs and PCBs (ng/g lipid) and detection frequency (%) in serum of domestic canines.

	%	All dogs (*n* = 51)	Control dogs (*n* = 36)	Hypothyroxinemic dogs (*n* = 15)
PBDEs				
BDE 47	92	208.81 (28.02–786.37)	135.24 (20.33–996.16)	401.30 (38.22–754.17)
BDE 99	88	100.90 (3.14–284.90)	38.16 (4.40–357.70)	177.54 (2.27–266.62)
BDE 100	87	15.28 (1.99–52.61)	11.04 (1.24–52.64)	23.71 (2.27–52.61)
BDE 66	79	12.40 (4.59–32.60)	10.92 (3.93–37.11)	17.72 (4.60–32.60)
BDE 49	73	3.37 (1.02–11.22)	2.69 (0.58–11.22)	4.90 (2.04–16.54)
BDE 153	69	7.38 (1.30–20.26)	9.40 (1.47–20.89)	6.05 (1.24–18.14)
BDE 28	58	2.56 (0.68–27.34)	1.45 (0.63–26.38)	14.30 (0.80–30.85)
BDE 85	58	5.54 (1.80–15.99)	5.74 (0.75–23.69)	4.30 (2.39–8.98)
BDE 136	46	2.48 (1.38–16.16)	2.27 (1.25–16.16)	6.51 (1.38–28.07)
BDE 154	38	3.11 (1.39–10.32)	3.11 (1.39–11.83)	3.39 (0.81–8.35)
BDE 183	35	1.96 (0.00–24.75)	0.60 (0.00–22.02)	9.68 (0.00–26.38)
PCBs				
PCB 101	100	91.38 (30.46–477.21)	77.00 (28.97–481.66)	223.60 (37.50–408.36)
PCB 11	100	42.81 (21.40–92.80)	46.82 (21.00–111.16)	40.40 (22.96–84.22)
PCB 118	100	122.56 (43.69–498.01)	90.83 (37.81–498.01)	224.24 (51.94–504.02)
PCB 132	100	20.37 (8.45–69.10)	17.52 (8.38–81.42)	37.27 (9.10–68.48)
PCB 138	100	37.49 (15.14–166.59)	31.30 (15.02–180.40)	70.29 (18.57–155.34)
PCB 153	100	15.95 (7.05–72.34)	13.71 (6.81–72.34)	33.47 (14.11–72.36)
PCB 52	98	65.11 (25.49–279.60)	57.93 (25.49–347.98)	136.40 (22.08–270.09)
PCB 66	98	217.48 (57.52–1003.35)	184.25 (64.24–1101.74)	507.15 (26.48–883.55)
PCB 84	98	10.90 (1.94–57.28)	8.49 (1.94–62.23)	25.60 (1.27–57.28)
PCB 95	96	10.23 (1.41–72.98)	8.77 (2.62–91.53)	41.04 (1.07–72.98)
PCB 28	90	9.34 (0.65–30.11)	8.92 (1.06–31.74)	12.26 (0.40–25.68)
PCB 149	75	8.19 (1.51–53.17)	5.28 (1.50–53.17)	26.81 (1.69–59.20)
PCB 91	65	2.10 (0.68–24.70)	1.78 (0.68–26.41)	2.60 (0.77–22.64)
PCB 135	65	8.53 (2.87–36.45)	7.80 (2.72–36.45)	10.51 (2.90–43.38)
PCB 180	60	6.92 (1.88–24.39)	6.87 (1.76–25.82)	6.92 (2.47–18.44)

PBDE and PCB congeners included in the logistic regression analysis are listed in order of decreasing frequency in Table 1. Odds ratios were determined for each PBDE and PCB congener, describing the relationship between an elevated concentration of the individual congener and the associated change in the odds of a canine patient having hypothyroxinemia (Table S5). For clarity of data interpretation, the odds ratios for BDE 100, BDE 99, PCB 11, PCB 132, PCB 138, PCB 149, PCB 153, PCB 52, PCB 84, and PCB 95 are presented corresponding to a 10 ng/g lipid increase in serum concentration because each of these congeners had a median concentration greater than 10 ng/g lipid for the hypothyroxinemic group. The odds ratios for BDE 47, PCB 101, PCB 118, and PCB 66 are presented corresponding to a 100 ng/g lipid increase in serum concentration because each of these congeners had a median greater than 100 ng/g lipid for the hypothyroxinemic group. The remaining PBDE and PCB congeners all had median values below 10 ng/g lipid in the hypothyroid group; their odds ratios are given as proportionate change in the odds of hypothyroxinemia associated with a 1 ng/g lipid increase in serum concentration. BDE 183 was the only congener that had a positive odds ratio close to being significantly different from 1.00 (1.05, 95% CI, 1.00–1.10, *p* = 0.068), indicating that although not significant this association was still unlikely to arise by chance under the assumptions of absence of bias and correctness of model specification. This odds ratio indicates that a 1 ng/g lipid increase in the serum concentration of BDE 183 was associated with a 5.0% increase in the odds of canine hypothyroxinemia.

## Discussion

In this study, serum concentrations of 14 PBDE and 23 PCB congeners were determined for 15 hypothyroxinemic and 36 control dogs presented to the UC Davis VMTH. PBDEs and PCBs were detected in all samples, with BDE 47, BDE 99, PCB 118, PCB 52, and PCB 66 having the highest mean and median concentrations of all the analyzed congeners. An exact logistic regression model was used to determine whether the concentration of suspect individual (or the sum of) congeners was significantly correlated with the incidence of canine hypothyroxinemia. This model was chosen for analysis of congener concentrations for two reasons: (1) exact logistic regression does not assume normality or equal variance and (2) it prevents small sample-size bias that can occur as a result of maximum likelihood estimation in a normal logistic regression model. Results from this study’s logistic regression analysis determined that current concentrations of PBDEs and PCBs in canines were not significantly associated with hypothyroxinemia. BDE -47, -99, -100, and -153 and PCB -28, -77, -95, and -101, were the only congeners from this study with reports of decreased serum T4 concentrations following exposure from other animal models that studied PBDE and PCB congeners individually (Desaulniers et al., 1997; Fernie et al., 2005; Khan et al., 2002). Scientific data remain to be inconsistent and inconclusive regarding the influence of PBDEs and PCBs on thyroid hormones (Zhao et al., 2015). However, most epidemiological studies in humans have identified associations between PBDE blood levels and thyroid hormone levels (Allen et al., 2016). Results of epidemiological studies are strengthened by rodent and mechanistic studies (Hallgren et al., 2001). Both individual and mixtures of PCBs can influence circulating thyroid hormone levels (Crofton, 2008). However, toxicity endpoints do not necessarily behave in a manner predicted by T4 levels in PCB treated animals. Bansal & Zoeller (2008) observed that PCBs decreased circulating T4 levels and induced neurotoxic effects; however, when the same level of T4 reduction was induced by propylthiouracil, a chemical that blocks thyroid hormone synthesis, the same downstream consequences were not observed (Bansal & Zoeller, 2008). This indicates that PCBs may exert cellular effects not predicted by the changes in circulating T4 (Bansal & Zoeller, 2008; WHO/UNEP, 2013). Similar results were observed in a study conducted by Yang et al. (2009), in which the authors did not find an association between abnormalities of dendritic growth in rats and altered thyroid hormone levels due to PCB exposure (Yang et al., 2009). This observation is not well understood, which makes interpreting hormone levels after PCB and PBDE exposure in a clinical, regulatory, or epidemiological setting difficult.

High canine metabolic rate is a potential reason as to why no association was found between the current PBDE and PCB levels and hypothyroxinemia. Studies have suggested that cats tend to accumulate persistent pollutants to a higher extent than dogs because dogs are metabolically better equipped at degrading these pollutants (Ruiz-Suarez et al., 2015; Venier & Hites, 2011). A recent study showed that environmental pollutants, like PCBs, have higher levels in dog food; thus, dogs have higher dietary intake of them. However, despite this, plasma levels of PCBs were higher in cats relative to dogs, consistent with dogs having better metabolizing capabilities (Ruiz-Suarez et al., 2015). Although PBDE exposure and metabolic studies in dogs are limited, the structural similarity between PCBs and PBDEs suggests high metabolic capacity for the latter as well (Voorspoels et al., 2006). Therefore, it is reasonable to believe that metabolism contributes a considerable role in the toxicokinetics and fate of PBDEs and PCBs, especially in species with higher metabolic capabilities. Mizukawa et al. (2016) detected hydroxylated PCBs (OH-PCBs) in the blood samples of both dogs and cats but found only a few OH-PCBs at extremely low levels in pet food products (Mizukawa et al., 2016). This suggested that OH-PCBs were formed *in vivo* from parent compounds. However, congener profiles of OH-PCBs differed between the two species. Tri- to penta-chlorinated OH-PCB congeners in the cat blood accounted for approximately 90% of the OH-PCBs. In contrast, hexa- to octa-chlorinated OH-PCBs (4OH-CB 199 and 4OH-CB 202) were greater in dog blood (Mizukawa et al., 2016). Kunisue & Tanabe (2009) observed similar results—an elevated composition of octa-chlorinated OH-PCBs in dogs and raccoon dogs—indicating that these metabolites could be retained in the blood for a long time compared to lower chlorinated OH-PCB congeners (Kunisue & Tanabe, 2009).

OH-PCBs are biologically active and formed by oxidative metabolism of PCBs by cytochrome P450 monooxygenases (Boas et al., 2006; Kunisue & Tanabe, 2009). Because of their structural resemblance to T4, OH-PCBs can disturb thyroid hormone homeostasis (Kunisue & Tanabe, 2009). Competitive binding assays have shown that OH-PCBs can bind to human serum transthyretin (TTR), a carrier protein of T4, and displace T4 from the serum TTR, suggesting that OH-PCB congeners have a stronger binding affinity for TTR than either T4 or the parent compounds (Boas, Feldt-Rasmussen & Main, 2012; Kunisue & Tanabe, 2009; Pearce & Braverman, 2009). Likewise, hydroxylated-PBDEs (OH-PBDEs) are structurally similar to thyroid hormones and may also disrupt thyroid homeostasis. In an *in vitro* competitive binding assay using human TTR and I-T4 as the displaceable radioligand PBDEs were able to compete with T4-TTR binding only after metabolic conversion, suggesting the important role for hydroxylation of PBDEs in their effects on thyroid homeostasis (Meerts et al., 2000). In addition, the degree of bromination of OH-PBDEs interferes with thyroid function through different activities on thyroid hormone receptors; lower-brominated OH-PBDEs bound weakly to thyroid hormone receptors and acted as agonists, whereas high-brominated compounds were more potent binders and acted as antagonists (Ren et al., 2013). Evidence of decreased thyroid hormones in offspring perinatally exposed to PCBs and their hydroxylated metabolites has also been reported in rats, sled dogs, polar bears, seals, and nesting eagles, indicating potential transfer of these metabolites across the placenta to the fetus with the possible consequence of altered thyroid hormone status in newborns (Boas, Feldt-Rasmussen & Main, 2012). Therefore, hydroxylation and halogenation of PBDEs and PCBs are important factors to consider in terms of thyroid toxicity.

Although not statistically significant, the data from this study found higher concentrations of PBDEs and PCBs in the hypothyroxinemic group compared to the control group, suggesting potential effects from pollutant exposure cannot be ruled out. The limitations of a small sample size may have contributed to the lack of significant associations observed. BDE 183 was the only congener showing near-significance (*p* = 0.068). BDE 183 is considered a marker compound for the octa-mix PBDE formulation. Evidence of BDE 183 in canine serum indicates that components of the octa-mix, which was principally used in molded parts of computers, televisions, car parts, and other products, are still entering the environment even though the manufacturing of the mix supposedly ceased in 2004 (EPA, 2014; Law et al., 2014). Debromination of large reservoirs of BDE 209 in soils and sediments may yield large quantities of lower-brominated congeners, including BDE 183 (Law et al., 2014). In *in vitro* root crude enzyme extracts from maize, ryegrass, and pumpkin, degradation of BDE 209 yielded congeners BDE 206, BDE 207, BDE 208 and BDE 183, as well as other lower brominated congeners down to BDE 7 (Huang et al., 2013). In juvenile common sole exposed to six BDE congeners including BDE 209, certain BDE congeners were detected in fish tissues that were not present in the spiked food, such as BDE 49, BDE 154, BDE 183, and BDE 202 (Munschy et al., 2011). In this case, the sole were able to metabolize BDE209 to yield lower brominated congeners (Law et al., 2014; Munschy et al., 2011). Similar results were also observed in studies involving rainbow trout and juvenile carp (Kierkegaard et al., 1999; Stapleton et al., 2004). Despite the phase-out of PBDE commercial mixes, breakdown of larger compounds has become an important consideration when assessing PBDE exposure sources.

**Table 2:** A comparison of PBDE concentrations in canine serum samples with concentrations reported in Indiana, Virginia, Pakistan, and Japan.

Country	USA (CA)	USA (IN)^a^	USA (VA)^b^	Pakistan^c^	Japan^d^^e^
	*N* = 51	*N* = 17	*N* = 20	*N* = 16	*N* = 17
BDE 17	0.54^f^	n.a.	n.a.	n.a.	n.a.
BDE 28	9.68	n.a.	0.0459	n.a.	n.a.
BDE 47	343.86	0.44	1.01	0.7	<0.0042
BDE 49	5.23	n.a.	n.d.	n.a.	n.a.
BDE 66	14.92	n.a.	n.d.	n.a.	n.a.
BDE 85	7.96	n.a.	0.0306	n.a.	n.a.
BDE 99	125.60	0.33	0.674	0.45	<0.0042
BDE 100	22.12	0.23	0.111	n.a.	<0.0042
BDE 153	9.71	0.11	2.01	0.35	<0.0042
BDE 154	4.30	0.098	0.0776	n.a.	<0.0042
BDE 183	7.65	n.a.	0.127	n.a.	<0.0042

**Notes.**

Concentrations are given as mean values and expressed in ng/g lipid.

a Venier & Hites (2011).

b EWG (2008).

c Ali et al. (2013).

d Mizukawa et al. (2016).

e Concentrations from canines in Japan are given in ng/g whole blood wet weight.

f Congener was only detected in one canine sample.

n.a. not applicable n.d. not detected

Several studies have documented the occurrence of PBDEs and PCBs in pet dogs (Tables 2 and 3). Results from the present study show higher PBDE and PCB levels in dogs in California relative to companion dogs in Indiana and Virginia in the USA, Pakistan, Japan, and Italy (Tables 2 and 3). In contrast, the mean (±SE) and median values of ΣM-PCBs (PCB-28, -52, -101, -138, -153, and -180) observed in dogs in Spain (67.1 ± 61.6; 50.10 ng/g lipid; (Ruiz-Suarez et al., 2015) were similar to the levels we observed in dogs in California (72.81 ± 70.66; 50.12 ng/g lipid). It is important to note that concentrations observed in Japan were measured in whole blood rather than serum; therefore, concentrations appear much lower. Generally, PBDE and PCB concentrations appeared much higher in the United States than most other countries. PBDEs have been used extensively in products in California until their proposed ban in 2003. Following the ban in 2004, two commercial formulations, penta-BDE and octa-BDE, were phased out of production in some US states after a voluntary agreement between the US EPA and the sole manufacturer of these products (Dodson et al., 2012). Despite the phase out of many PBDEs used in industry, these compounds persist in our environment due to their resistance to biodegradation (Bradman et al., 2014; Dodson et al., 2012; Whitehead et al., 2015). Prior to the ban, California mainly used penta-BDE mixtures, comprised mainly of BDE-47 and -99 (>70%), with smaller contributions from BDE-100, BDE-153, and BDE-154 (La, Hale & Harvey, 2006; Stapleton et al., 2012). One epidemiological study assessed sera PBDEs in mothers and their children from the Bay Area of California and found that BDE-47, -99, -100, -153 made up ∼90% of serum PBDE content (Eskenazi et al., 2013) consistent with the composition of flame retardants. Our data agrees with these trends with the Bay Area having the highest concentrations of BDE-47, -99, and -100. Our results also agree with a recent study investigating PBDE congener profiles in felines from California (Walter et al., 2017). In contrast, a feline study conducted in Sweden identified BDE209 and BDE207 as the most prevalent BDE congeners with BDE99 accounting for only 13% total body burden, compared to 44% in a US population (Kupryianchyk et al., 2009). Therefore, regional variability in PBDE congener profile will likely influence the collinearity of congeners in human, feline, or canine samples. This extends to other biological as well as environmental matrices. A study by Zota et al. (2008) noted that PBDE concentrations measured in house dust, and serum and breast milk of humans were much higher in US samples compared to European samples. This study further researched regional variation within the US and found the highest levels of PBDEs occurring in the Western region, including California. The observed high levels of PBDEs appear to be because of the stringent fire safety laws enacted in the state of California that eventually impacted products throughout the US Reports have shown that about half of the total PBDEs and 95% of the penta-BDE used worldwide were consumed in North America (Ali et al., 2013). Our data showed that depending on the congener, PBDE concentrations in dogs were approximately 5.5 to 10 times higher compared to levels detected in humans. This is in agreement with studies in cats; young cats had up to 20 times greater PBDE levels than the median values reported for US adults (Dye et al., 2007a; Dye et al., 2007b). The major exposure route for PBDEs in pets is thought to be from household dust (Guo et al., 2012) as pets spend most of their time on the floor or in areas where dust tends to accumulate. Because of the pets’ intensive grooming behaviors, dust ingestion may also contribute to the elevated PBDE congener levels found in dogs and cats compared with those reported in humans. This also suggests dogs as suitable sentinels for infants, who often come in contact with environmental contaminants through their frequent hand-to-mouth activity and exploratory behavior.

**Table 3:** A comparison of PCB concentrations in canine serum samples with concentrations reported in Japan and Italy.

Country	USA (CA)	Japan^a^^c^	Italy^b^
	*N* = 51	*N* = 17	*N* = 91
PCB 28	12.82	<0.0074	n.a.
PCB 52	130.75	<0.0074	2.48
PCB 101	187.46	0.00043	2.87
PCB 138	69.68	0.0012	6.37
PCB 153	31.82	0.0027	5.21
PCB 180	10.06	0.0032	16.01
PCB 77	6.25^d^	n.a.	0.52
PCB 118	209.09	0.00073	2.37
PCB 95	30.80	<0.0074	n.a.
PCB 149	23.95	<0.0074	n.a.

**Notes.**

Concentrations are given as mean values and expressed in ng/g lipid.

a Mizukawa et al. (2016).

b Storelli et al. (2009).

c Concentrations from canines in Japan are given in ng/g whole blood wet weight.

d Congener was only detected in one canine sample.

n.a. not applicable

PBDE and PCBs levels have reportedly declined in California and a recent study on Arctic foxes from Norway reported decreased PBDE and PCB concentrations from 1997 to 2013. The observed decline in concentrations of these pollutants observed in humans and wildlife suggest a positive impact of governmental policies (Andersen et al., 2015; Guo et al., 2016). Despite the declining levels of persistent organic pollutants in California, levels of PBDEs and PCBs in human breast milk were still higher in samples from California than samples reported from China and New Zealand (Guo et al., 2016). In 2012, the European Food Safety Authority (EFSA) reported that current dietary exposure to BDE 99 raises potential health concerns (EFSA, 2011). Results from this study showed 88% detection frequency for BDE99 in dog serum, with the highest concentration similar to reports in other states and countries (Table 2). Exposure to these pollutants has been linked to cancers, neurobehavioral and developmental disorders in addition to thyroid disease (Carpenter, 2006; Kim et al., 2014). Given the current levels of PBDEs and PCBs, potential health concerns other than thyrotoxicity, such as neurotoxicity, estrogenicity, and carcinogenicity should also be investigated.

In conclusion, detected levels of PBDEs and PCBs in domestic canines from this study were not significantly associated with thyroid hormone disruption. Future exposure studies involving canine models should take into account toxicokinetic parameters, such as absorption, bioaccumulation, metabolism, and excretion, in order to provide a better understanding of the relationship between PBDE and PCB exposure and adverse effects on animals and humans. Should future research find evidence that PBDEs and PCBs act as endocrine disrupting chemicals by disrupting thyroid function in pets, this would just add credence to the hypothesis that such an endocrinopathy could occur in humans as well.

##  Supplemental Information

10.7717/peerj.3780/supp-1 Supplemental Information 1 Supplemental Tables

10.7717/peerj.3780/supp-2 Data S1 Raw data

## Abbreviations

CI: Confidence intervals
OH-PBDEs: Hydroxylated polybrominated diphenyl ethers
OH-PCBs: Hydroxylated polychlorinated biphenyls
OR: Odds ratio
PBDEs: Polybrominated diphenyl ethers
PCBs: Polychlorinated biphenyls
QC: Quality Control
T4: Thyroxine
TC: Total cholesterol
TG: Total triglycerides
TL: Total lipids
VMTH: Veterinary Medical Teaching Hospital

## Additional Information and Declarations

### Data Availability

The following information was supplied regarding data availability: The raw data has been supplied as a Supplementary File.
