# Supplementary material for: Comparison of polybrominated diphenyl ethers (PBDEs) and polychlorinated biphenyls (PCBs) in the serum of hypothyroxinemic and euthyroid dogs
Source: PeerJ. 2017 Sep 12;5:e3780. doi: 10.7717/peerj.3780 (PMC5600179; doi:10.7717/peerj.3780)
Supplement: Supplemental Information 1 [file peerj-05-3780-s001.docx]

**Supplementary Data**

Table S1. Population demographics of canines included in the present study including diagnosis of hypothyroidism, gender, gonadal status, age (years), weight (kg), breed, and size.

| **ID** | **Diagnosis of Hypothyroidism** | **Gender** | **Neutered/ Spayed** | **Age** | **Weight** | **Breed** | **Size*** |
| --- | --- | --- | --- | --- | --- | --- | --- |
| 1 |  | Female | Spayed | 10 | 6.3 | Chihuahua | Small |
| 3 |  | Male |  | 13.5 | 6.8 | Bichon Frise | Small |
| 4 | yes | Female | Spayed | 12 | 24.6 | Mix Staffordshire Terrier/Rottweiler | Medium |
| 6 | yes | Female | Spayed | 11 | 21.1 | Springer Spaniel | Medium |
| 7 |  | Male | Castrated | 7 | 2.9 | Pomeranian | Small |
| 8 |  | Female | Spayed | 7 | 31.2 | Mixed breed | Large |
| 10 | yes | Female | Spayed | 11 | 19.6 | Border Collie mix | Medium |
| 12 | yes | Male | Castrated | 10 | 17.3 | Dachshund | Medium |
| 13 | yes | Male | Castrated | 10 | 33.8 | Labrador | Large |
| 14 | yes | Male | Castrated | 8 | 40.7 | Briard | Large |
| 15 |  | Male | Castrated | 5 | 62.2 | Rottweiler | Large |
| 16 |  | Male |  | 7 | 47.6 | Flat Coated Retriever | Large |
| 17 | yes | Female | Spayed | 11.5 | 22.2 | Border Collie | Medium |
| 18 |  | Male |  | 9 | 25.4 | Australian Shepherd | Medium |
| 19 |  | Male | Castrated | 7 | 13 | Miniature Schnauzer | Small |
| 23 |  | Male | Castrated | 8 | 7 | Bichon Frise | Small |
| 24 |  | Female | Spayed | 10 | 41.8 | Mix Anatolian Shepherd/Great Pyrenees | Large |
| 25 | yes | Female | Spayed | 12 | 12.7 | Shetland Sheepdog | Small |
| 26 |  | Female | Spayed | 6 | 57 | Chesapeake Bay Retriever | Large |
| 27 |  | Female | Spayed | 8 | 17.2 | Beagle | Small |
| 28 | yes | Female | Spayed | 12 | 29 | Labrador | Large |
| 29 |  | Female | Spayed | 6 | 26.5 | Pit Bull Terrier | Medium |
| 30 | yes | Male | Castrated | 9 | 32.7 | Labrador | Large |
| 31 |  | Male | Castrated | 14 | 10.3 | Mix Terrier Poodle | Small |
| 32 | yes | Male | Castrated | 5 | 38.4 | Mix German Shepherd/Pit Bull | Large |
| 33 |  | Male | Castrated | 7 | 25.4 | English Bulldog | Medium |
| 34 |  | Male | Castrated | 5 | 67 | Great Pyrenees | Large |
| 35 | yes | Male | Castrated | 9 | 44.3 | Pit Bull Terrier | Medium |
| 36 |  | Male | Castrated | 12 | 19.1 | Mix Welsh Corgie, Pembroke | Small |
| 37 | yes | Female | Spayed | 10 | 49.1 | Great Pyrenees | Large |
| 39 | yes | Male | Castrated | 7 | 46.5 | German Shepherd | Large |
| 40 | yes | Female | Spayed | 12 | 20.6 | Australian Shepherd | Medium |
| 41 |  | Male | Castrated | 10 | 38 | Labrador | Large |
| 42 |  | Male | Castrated | 6 | 18.5 | Border Collie/X | Medium |
| 43 |  | Male | Castrated | 12 | 34.3 | Husky | Medium |
| 44 |  | Female | Spayed | 1.5 | 29.5 | Labrador - Mix | Large |
| 45 |  | Female | Spayed | 5 | 5.3 | Schnauzer | Small |
| 46 |  | Male |  | 9 | 11.9 | Cocker Spaniel | Small |
| 47 |  | Male |  | 4.5 | 40.7 | Labrador | Large |
| 48 |  | Male | Castrated | 10 | 39 | Labrador | Large |
| 49 |  | Male | Castrated | 13 | 11.7 | Shetland Sheepdog | Small |
| 50 |  | Female | Spayed | 7 | 27 | Labrador | Large |
| 51 |  | Male | Castrated | 15 | 24.8 | Australian Cattledog | Medium |
| 52 |  | Male | Castrated | 10 | 31 | Labrador | Large |
| 53 |  | Male | Castrated | 9 | 38.9 | Airedale Terrier | Medium |
| 54 |  | Male | Castrated | 6 | 38 | Pit Bull Terrier | Medium |
| 56 |  | Male | Castrated | 2 | 29.7 | Labrador | Large |
| 57 |  | Male | Castrated | 4 | 66.5 | Saint Bernard | Large |
| 58 |  | Female | Spayed | 12 | 20.2 | Mix Siberian Husky-Lab | Medium |
| 59 |  | Female | Spayed | 12 | 23.6 | Mix-Australian Cattle Dog | Medium |
| 60 |  | Male | Castrated | 12 | 32 | Pit Bull Terrier | Medium |

*Size was determined by breed as indicated by *The Kennel Club* (kennelclub.org.uk).

Table S2. Lower limits of detection (LOD) and quantification (LOQ), correlation coefficient from regression weight 1/x, and % Recovery ^a^ of quality control (QC) samples for each PBDE and PCB congener from three repetitions.

|  |  |  |  | % Recovery | | |
| --- | --- | --- | --- | --- | --- | --- |
| Congeners | LOD (ng/mL) | LOQ (ng/mL) | Correlation Coefficient (r) | QC1 | QC2 | QC3 |
| **PBDEs** |  |  |  |  |  |  |
| BDE 100 | 0.0175 | 0.0583 | 0.9977 | 97.67 ± 4.46 | 98.78 ± 10.04 | 107.59 ± 10.62 |
| BDE 136 | 0.0003 | 0.0009 | 0.9987 | 105.93 ± 20.74 | 91.55 ± 2.54 | 107.50 ± 12.23 |
| BDE 153 | 0.0003 | 0.0009 | 0.9979 | 107.55 ± 11.66 | 89.64 ± 0.90 | 103.99 ± 13.62 |
| BDE 154 | 0.0118 | 0.0393 | 0.9986 | 94.59 ± 12.10 | 95.43 ± 12.17 | 101.99 ± 13.51 |
| BDE 17 | 0.0045 | 0.0149 | 0.9980 | 88.67 ± 11.82 | 106.16 ± 7.89 | 114.66 ± 6.71 |
| BDE 183 | 0.0035 | 0.0118 | 0.9981 | 113.03 ± 1.19 | 94.11 ± 16.47 | 106.67 ± 15.61 |
| BDE 28 | 0.0095 | 0.0316 | 0.9975 | 102.86 ± 18.77 | 100.72 ± 8.03 | 113.42 ± 9.64 |
| BDE 47 | 0.0190 | 0.0635 | 0.9980 | 100.52 ± 3.26 | 96.78 ± 15.91 | 110.67 ± 15.68 |
| BDE 49 | 0.0291 | 0.0969 | 0.9988 | 110.38 ± 9.79 | 99.58 ± 6.88 | 108.64 ± 12.88 |
| BDE 52 | 0.0228 | 0.0761 | 0.9985 | 90.78 ± 13.83 | 97.86 ± 3.08 | 109.09 ± 11.27 |
| BDE 66 | 0.0540 | 0.1799 | 0.9987 | 107.12 ± 4.90 | 111.51 ± 6.12 | 109.83 ± 9.03 |
| BDE 85 | 0.0311 | 0.1037 | 0.9974 | 94.74 ± 15.73 | 105.64 ± 1.86 | 110.43 ± 6.92 |
| BDE 95 | 0.0128 | 0.0428 | 0.9996 | 98.20 ± 11.83 | 97.77 ± 8.45 | 107.59 ± 11.10 |
| BDE 99 | 0.0156 | 0.0520 | 0.9983 | 99.46 ± 11.43 | 100.73 ± 4.43 | 105.79 ± 16.53 |
| **PCBs** |  |  |  |  |  |  |
| PCB 101 | 0.0109 | 0.0365 | 0.9950 | 110.89 ± 7.00 | 101.51 ± 13.18 | 108.82 ± 3.44 |
| PCB 11 | 0.0025 | 0.0083 | 0.9937 | 112.44 ± 3.87 | 110.56 ± 10.22 | 115.87 ± 5.29 |
| PCB 118 | 0.0381 | 0.1271 | 0.9974 | 100.82 ± 9.87 | 106.33 ± 13.48 | 106.55 ± 10.96 |
| PCB 131 | 0.1518 | 0.5058 | 0.9954 | 105.78 ± 12.02 | 97.59 ± 10.89 | 107.56 ± 9.23 |
| PCB 132 | 0.0385 | 0.1282 | 0.9982 | 112.84 ± 5.98 | 95.07 ± 3.72 | 105.75 ± 12.15 |
| PCB 135 | 0.0468 | 0.1561 | 0.9955 | 98.70 ± 17.73 | 103.87 ± 15.26 | 112.15 ± 7.76 |
| PCB 136 | 0.0435 | 0.1448 | 0.9959 | 97.50 ± 10.95 | 105.88 ± 5.29 | 111.90 ± 8.71 |
| PCB 138 | 0.0195 | 0.0651 | 0.9959 | 100.27 ± 11.62 | 91.23 ± 11.91 | 107.71 ± 10.64 |
| PCB 149 | 0.0524 | 0.1746 | 0.9982 | 94.42 ± 2.51 | 91.59 ± 7.94 | 106.64 ± 8.99 |
| PCB 153 | 0.0386 | 0.1288 | 0.9970 | 104.90 ± 7.19 | 96.08 ± 6.15 | 109.88 ± 7.32 |
| PCB 174 | 0.0325 | 0.1083 | 0.9961 | 93.84 ± 18.81 | 103.28 ± 7.48 | 106.68 ± 9.93 |
| PCB 175 | 0.0286 | 0.0952 | 0.9980 | 107.67 ± 8.74 | 91.83 ± 10.75 | 106.15 ± 11.19 |
| PCB 176 | 0.0114 | 0.0380 | 0.9970 | 106.40 ± 16.23 | 97.79 ± 2.28 | 108.30 ± 10.01 |
| PCB 180 | 0.0239 | 0.0796 | 0.9976 | 102.82 ± 15.66 | 96.73 ± 8.75 | 109.86 ± 8.73 |
| PCB 196 | 0.0037 | 0.0122 | 0.9976 | 99.89 ± 13.34 | 90.35 ± 11.10 | 108.88 ± 9.22 |
| PCB 202 | 0.0891 | 0.2971 | 0.9987 | 103.26 ± 8.50 | 103.08 ± 17.96 | 105.97 ± 8.64 |
| PCB 28 | 0.0058 | 0.0195 | 0.9943 | 105.53 ± 13.36 | 100.99 ± 16.20 | 117.99 ± 3.21 |
| PCB 52 | 0.0026 | 0.0088 | 0.9934 | 104.34 ± 3.59 | 95.58 ± 12.89 | 115.79 ± 2.47 |
| PCB 66 | 0.0074 | 0.0247 | 0.9947 | 104.31 ± 14.56 | 95.71 ± 12.86 | 108.86 ± 9.85 |
| PCB 77 | 0.0397 | 0.1324 | 0.9949 | 99.23 ± 14.74 | 101.54 ± 9.37 | 112.85 ± 6.07 |
| PCB 84 | 0.0104 | 0.0346 | 0.9940 | 103.80 ± 11.01 | 103.78 ± 4.36 | 112.15 ± 9.22 |
| PCB 91 | 0.0104 | 0.0346 | 0.9937 | 103.12 ± 12.61 | 98.92 ± 10.27 | 109.95 ± 6.88 |
| PCB 95 | 0.0054 | 0.0180 | 0.9941 | 99.18 ± 12.40 | 97.53 ± 5.00 | 111.49 ± 7.71 |

^a^ % Recovery for each congener is given as a mean value (± standard error; SE).

Table S3. Comparison of population demographics and characteristics between canine control and hypothyroxinemic study groups^a, b, c^.

| Variable | Control (*36*) | Hypothyroxinemia (*15*) | p-value |
| --- | --- | --- | --- |
| **Age – years** | | | |
| Mean ± SE  Median (5-95 pctl) | 8.38 ± 3.35  8 (3.5 – 13.6) | 9.97 ± 2.07  10 (6.4 – 12) | 0.093 |
| **Sex – percent** | | | |
| Male (*32*)  Female (*19*) | 69%  31% | 47%  53% | 0.203 |
| **Weight – kg** | | | |
| Mean ± SE  Median (5-95 pctl) | 28.54 ± 17.09  26.75 (6.05 – 63.28) | 30.17 ± 11.57  29 (15.92 – 47.28) | 0.536 |
| **Size – percent** | | | |
| Small + Medium (*30*)  Large (*21*) | 61%  39% | 53%  47% | 0.757 |
| **Total Lipids (g/ml)** | | | |
| Mean ± SE  Median (5-95 pctl) | 0.0080 ± 0.0025  0.0075 (0.0049-0.0125) | 0.0076 ± 0.0016  0.0073 (0.0053-0.0121) | 0.767 |

^a^ Age and weight are given as mean (± standard error; SE) and median concentrations (range: 5^th^ – 95^th^ percentiles).

^b^ Sex and size are given as percentages.

^c^ Total lipids are given as mean concentrations (± standard error; SE).

Table S4. Coefficients from Least Squares Linear Regression Analysis to evaluate the relationship between concentrations of total lipids and each PBDE and PCB congener in canine serum samples.

| Congener | Least Squares Linear Regression Coefficient (95% CI) | p-value |
| --- | --- | --- |
| PBDEs |  |  |
| BDE 100 | 9.35 (-10.69 – 29.39) | 0.353 |
| BDE 136 | -1.28 (-8.36 – 5.80) | 0.719 |
| BDE 153 | 5.68 (-1.66 – 13.02) | 0.126 |
| BDE 154 | -0.33 (-3.45 – 2.78) | 0.830 |
| BDE 183 | -0.63 (-12.13 – 10.88) | 0.913 |
| BDE 28 | 1.10 (-8.83 – 11.04) | 0.825 |
| BDE 47 | 110.94 (-206.35 – 428.23) | 0.486 |
| BDE 49 | 3.62 (-0.76 – 8.00) | 0.103 |
| BDE 66 | -2.57 (-11.70 – 6.55) | 0.573 |
| BDE 85 | 4.55 (-3.05 – 12.16) | 0.235 |
| BDE 99 | 54.34 (-55.52 – 164.19) | 0.325 |
|  |  |  |
| PCBs |  |  |
| PCB 101 | 48.52 (-116.86 – 213.90) | 0.558 |
| PCB 11 | 21.67 (-6.64 – 49.99) | 0.130 |
| PCB 118 | 28.15 (-135.73 – 192.03) | 0.731 |
| PCB 132 | -0.029 (-23.43 – 23.38) | 0.998 |
| PCB 135 | -0.91 (-20.41 – 18.59) | 0.926 |
| PCB 138 | 16.83 (-38.96 – 72.62) | 0.547 |
| PCB 149 | 6.49 (-18.88 – 31.86) | 0.609 |
| PCB 153 | -0.81 (-26.09 – 24.48) | 0.949 |
| PCB 180 | -4.30 (-11.08 – 2.48) | 0.209 |
| PCB 28 | 6.63 (-3.33 – 16.59) | 0.187 |
| PCB 52 | 34.16 (-74.65 – 142.98) | 0.531 |
| PCB 66 | 166.94 (-210.47 – 544.36) | 0.378 |
| PCB 84 | 7.50 (-14.94 – 29.94) | 0.505 |
| PCB 91 | -1.92 (-10.62 – 6.78) | 0.659 |
| PCB 95 | 6.68 (-24.44 – 37.80) | 0.668 |

Table S5. Odds ratios^a, b^ of canine hypothyroxinemia by serum concentrations of PBDE and PCB congeners^c^.

| Congener | Odds Ratio (95% CI) | p-value |
| --- | --- | --- |
| PBDEs |  |  |
| BDE 100 | 1.13 (0.85 – 1.50) | 0.413 |
| BDE 136 | 1.04 (0.97 – 1.12) | 0.262 |
| BDE 153 | 0.97 (0.90 – 1.05) | 0.471 |
| BDE 154 | 0.99 (0.83 – 1.18) | 0.827 |
| BDE 183 | 1.05 (1.00 – 1.10) | 0.068* |
| BDE 28 | 1.04 (0.98 – 1.11) | 0.184 |
| BDE 47 | 1.04 (0.87 – 1.24) | 0.657 |
| BDE 49 | 1.06 (0.94 – 1.21) | 0.306 |
| BDE 66 | 1.01 (0.96 – 1.07) | 0.597 |
| BDE 85 | 0.94 (0.85 – 1.04) | 0.256 |
| BDE 99 | 1.02 (0.97 – 1.08) | 0.442 |
| **ΣPBDEs** | 1.00 (1.00 – 1.00) | 0.559 |
|  |  |  |
| PCBs |  |  |
| PCB 101 | 1.06 (0.76 – 1.47) | 0.750 |
| PCB 11 | 0.94 (0.77 – 1.15) | 0.552 |
| PCB 118 | 1.03 (0.75 – 1.43) | 0.840 |
| PCB 132 | 1.05 (0.84 – 1.31) | 0.695 |
| PCB 135 | 1.00 (0.98 – 1.03) | 0.685 |
| PCB 138 | 1.02 (0.92 – 1.12) | 0.726 |
| PCB 149 | 1.03 (0.82 – 1.30) | 0.787 |
| PCB 153 | 1.08 (0.88 – 1.33) | 0.460 |
| PCB 180 | 0.99 (0.92 – 1.06) | 0.687 |
| PCB 28 | 0.97 (0.91 – 1.03) | 0.337 |
| PCB 52 | 1.01 0.96 – 1.06) | 0.839 |
| PCB 66 | 1.00 (0.86 – 1.16) | 0.993 |
| PCB 84 | 1.06 (0.83 – 1.37) | 0.632 |
| PCB 91 | 0.99 (0.93 – 1.05) | 0.804 |
| PCB 95 | 1.04 (0.87 – 1.24) | 0.680 |
| **ΣPCBs** | 1.00 (1.00 – 1.00) | 0.873 |

^a^ Odds ratios for BDE-100, -99, PCB-11, -132, -138, -149, -153, -52, -84, and -95 represent the increase in odds associated with a 10 ng/g lipid increase in congener concentration.

^b^ Odds ratios for BDE 47, PCB-101, -118, and -66 represent the increase in odds associated with a 100 ng/g lipid increase in congener concentration.

^c^ The logistic regression analyses of the relationship between serum concentrations of PBDE and PCB congeners and canine hypothyroxinemia controls for age of canine study participants.

* p-value close to significance (≤ 0.05)
